# Automated Assessment of Manual Dexterity Using a Sensorized Nine-Hole Peg Test Board: Reproducibility and Innovative Quantitative Metrics

**DOI:** 10.3390/s26113497

**Published:** 2026-06-01

**Authors:** Monica Biggio, Erica Grange, Federica Di Antonio, Ludovico Pedullà, Jessica Podda, Simone Denei, Costanza Iester, Giampaolo Brichetto, Marco Bove, Laura Bonzano

**Affiliations:** 1 Department of Neuroscience, Rehabilitation, Ophthalmology, Genetics, Maternal and Child Health (DINOGMI), University of Genoa, 16132 Genoa, Italy; monica.biggio@unige.it (M.B.); laura.bonzano@unige.it (L.B.); 2 neuroBRITE Research Center, Italian Multiple Sclerosis Foundation, 16149 Genoa, Italy; erica.grange@fismets.it (E.G.); federica.diantonio@fismets.it (F.D.A.); ludovico.pedulla@aism.it (L.P.); jessica.podda@aism.it (J.P.); giampaolo.brichetto@aism.it (G.B.); 3 Department of Informatics, Bioengineering, Robotics and Systems Engineering (DIBRIS), University of Genoa, 16145 Genoa, Italy; 4 Department of Experimental Medicine (DIMES), University of Genoa, 16132 Genoa, Italy; costanza.iester@edu.unige.it; 5 Ermit s.r.l., 16152 Genoa, Italy; simone@ermit.it; 6 IRCCS Azienda Ospedaliera Metropolitana (AOM), 16132 Genoa, Italy

**Keywords:** nine-hole peg yest, sensorized pegboard, digital assessment, manual dexterity, concurrent validity, test–retest reliability

## Abstract

The Nine-Hole Peg Test (9-HPT) is widely used in clinical settings but typically relies on the assessor’s expertise to record execution time. Here, we propose a novel sensorized 9-HPT capable of automatically measuring total execution time and, importantly, extracting a set of newly defined temporal parameters that enable a more detailed and objective characterization of task performance. We first demonstrated concurrent validity between the sensorized 9-HPT and stopwatch-based measurements recorded by an assessor (ρ always > 0.98; *p* < 0.001) in healthy participants. Agreement between methods was further supported by the Bland–Altman analysis, showing negligible bias and narrow limits of agreement. A linear mixed-effects model confirmed no systematic differences between methods but showed significant differences between dominant and non-dominant hands. Test–retest reliability of total completion time, assessed across two sessions, was good for both the dominant (ICC = 0.81) and non-dominant (ICC = 0.74) hands. The newly introduced temporal parameters also showed significant reliability (ICC = 0.73–0.78), particularly for the dominant hand. Overall, these findings support the reliability of the sensorized 9-HPT for standard outcome measures and highlight its added value in providing novel temporal metrics that more precisely capture the different phases of task execution.

## 1. Introduction

Manual dexterity is defined as the ability to perform oriented, coordinated movement with both hand and fingers and to manipulate objects [1,2].

The Nine-Hole Peg Test (9-HPT), originally introduced by Kellor and colleagues [3] in 1971, is a standardized test for manual dexterity, with task completion time as the primary outcome variable. It is used in populations with hand dysfunction due to hand injuries, chronic and neurological diseases (stroke [4,5,6], multiple sclerosis [7,8,9], Parkinson’s disease [10] or Charcot–Marie–Tooth disease [3]). Now, it is the established gold standard for evaluating fine manual dexterity in several clinical and research areas [11].

The test consists of a plastic board featuring a shallow circular tray containing the pegs and a nine-hole pegboard on the other side that must be filled and then emptied again as quickly as possible. It assesses fine motor dexterity for both the dominant and the non-dominant hand by recording the total time, in seconds, required for a participant to execute the task [2]. Notably, 9-HPT scores were found to correlate with other upper limb measures and activities of daily living [12]. However, approximately 53% of the variance in the 9-HPT score was explained by muscle strength, tactile sensitivity of the thumb, and presence of intention tremor [13]. In fact, the total time parameter may not be descriptive of the quality of the movement performed in the recorded time, nor of which phase of the movement a person may be impaired in, whether it be the selection of pegs or their manipulation [14].

Furthermore, one could suggest that the 9-HPT engages several cognitive processes, as the task requires motor planning capability to fill and empty the nine holes as quickly as possible. Although the current literature does not specifically address the cognitive component involved in performing the 9-HPT, insights can be drawn from studies with different primary objectives, whose findings offer interesting evidence of a potential association between the 9-HPT and cognitive functioning. For instance, the 9-HPT score has been correlated with information processing speed in people with multiple sclerosis [15], which is most often altered in this clinical population [16], and poorer performance on the 9-HPT has been linked to decreased resting state functional connectivity within executive networks [17]. Additionally, a recent study found an involvement of cognitive/prefrontal areas during the 9-HPT [18]. Therefore, this suggests that manual dexterity is the ability to make coordinated hand and finger movements to grasp and manipulate objects, but with a broader clinical meaning that goes beyond the mere motor aspect.

Notably, manual stopwatch-based timing may introduce operator-dependent variability due to reaction time [19] and, importantly, restricts the assessment to total task duration without enabling the extraction of finer-grained temporal parameters, which may offer a more comprehensive characterization of task performance.

Several approaches have been proposed in this framework to improve 9-HPT performance measurement. Some studies have explored fully digital or virtual implementations of the test, including tablet-based applications and virtual reality environments, which enable automated data collection but often modify the original task structure [20,21,22]. Others have relied on video recordings of task execution [23,24]. In parallel, sensorized versions of the 9-HPT have been developed to replicate the experimenter’s measurement while introducing automation elements [25,26]. Although some of these approaches have enabled the extraction of additional parameters to better characterize task execution, they frequently rely on systems that alter the physical properties or ecological validity of the original test [22,23,24].

Here, we propose the use of a novel platform based on the 9-HPT for an automated assessment of manual dexterity. The platform has a sensor-equipped board that automatically detects peg insertion and removal and is connected via Bluetooth to an Android app. This device preserves the key characteristics of the standard 9-HPT, including its portability and the physical features of the pegs and board, while enabling automated measurement. It provides an objective assessment of total execution time and, importantly, allows the extraction of additional temporal parameters beyond the overall duration, such as individual insertion and removal times and the intervals between successive peg insertions and removals. These metrics enable a more comprehensive and precise characterization of manual dexterity.

This study aimed to describe the sensorized 9-HPT device and establish its reliability and reproducibility in a cohort of healthy participants, including the assessment of agreement between the standard and automated versions of the test, as well as test–retest reliability. It also aimed to introduce and characterize a set of novel temporal parameters enabled by the sensorized device and evaluate their consistency across two sessions.

## 2. Materials and Methods

### 2.1. Participants

The total sample size was determined using the most conservative estimate.

To assess concurrent validity, an a priori power analysis was conducted using G*Power (v. 3.1.9.7), presuming a bivariate normal correlation model. We anticipated a minimum correlation of r = 0.7 between the two approaches, as values around 0.7 are commonly considered to indicate a strong association between measurements [27].

A one-tailed test was performed since we hypothesized that both approaches should yield time estimates in the same direction. We fixed the Type I error rate (α) at 0.01 and Type II error rate (β) at 0.05 (power = 0.95). A minimum of 24 healthy participants would be necessary under these conditions.

A separate power analysis was conducted to estimate the required sample size for assessing test–retest reliability. A priori sample size estimation using the ICC.Sample.Size R package [28] indicated that 36 participants were required (α = 0.05, one-tailed; power = 0.90), assuming a minimum acceptable ICC of 0.50 and an expected ICC of 0.75 corresponding to the boundary between poor and at least moderate reliability [29]. ICCs were calculated based on a two-way mixed-effects model with absolute agreement for the average of measurements.

Finally, 36 participants were enrolled for both the concurrent analysis between standard measurements and the sensorized device and the test–retest reliability in two separate sessions.

The participants were adults from 18 to 60 years, with no history of neurological disorders or peripheral nerve conditions. Individuals with orthopedic conditions or injuries that may affect upper limb or hand function, including fractures, tendon damage, or arthritis, were excluded. No other medical or psychiatric conditions that could interfere with upper limb mobility or task performance was reported. After 1–2 weeks, the participants were contacted again to undergo the retest phase.

Participants provided written informed consent before taking part in the study. The study was approved by the local ethics committee (Comitato Etico per la Ricerca di Ateneo—CERA, N. 2025.55 del Comitato Etico per la Ricerca di Ateneo) and was conducted in accordance with the Declaration of Helsinki.

### 2.2. Sensorized Platform for Automated 9-HPT

**Figure 1:**
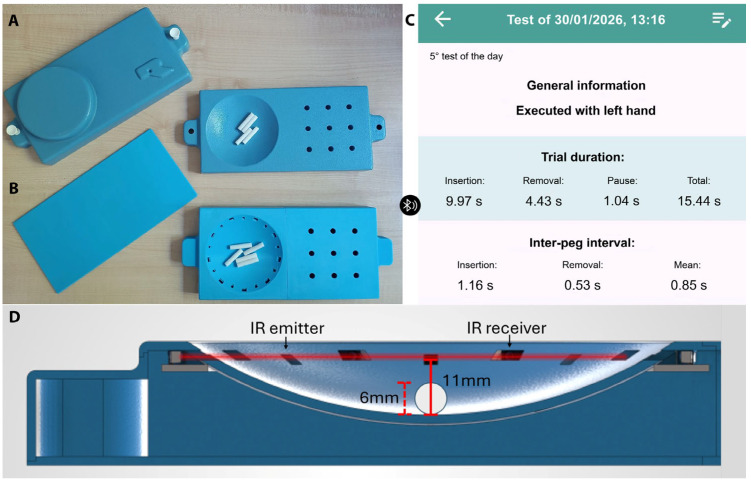
Figure shows (**A**) the standard test, commonly used for assessing manual dexterity in clinical settings; (**B**) the newly developed sensorized board, capable of detecting peg insertion and removal events; (**C**) the dedicated app, connected via Bluetooth to the sensorized device, used to manage and display the collected data; (**D**) section of the tray (maximum depth: 11 mm) with a representative peg (white disk, height: 6 mm), where the holes correspond to the infrared emitters and receivers composing the sensing array (one infrared beam is shown as an example).

The platform is based on a 3D-printed 9-HPT pegboard that complies with the original test measures design proposed by Mathiowetz [30,31], further integrated with the sensors (Figure 1).

The system comprises two distinct sensing arrays: one designed to monitor interactions within the tray area, and the other dedicated to detecting peg insertions into the holes.

The tray sensing system is designed to detect interactions occurring when either a hand or a peg enters or leaves the tray region. The tray sensing array is composed of independent infrared emitters and receivers (TSOP75438TR) arranged in a circular geometry, with each emitter facing its corresponding receiver. This configuration forms an optical grid capable of detecting the presence of obstacles within the tray area. The geometry of each slot was optimized to provide optical isolation between adjacent emitter–receiver pairs, ensuring that each emitter is detected only by its corresponding receiver and not by neighboring receivers.

Each of the nine holes is equipped with a reflective optical sensor (Vishay TCRT1010) with transistor output. This device integrates an infrared emitter and a phototransistor arranged side by side to measure the proximity of a reflective object positioned in front of the sensor. The flat white bottom surface of the peg provides a highly suitable reflective target. The sensor exhibits its peak response at approximately 1 mm, in order to detect the presence or absence of a peg and is particularly well suited to discriminate between a correctly inserted peg and an improperly seated one.

All sensors are sampled at 100 Hz, resulting in a maximum detection latency of 10 ms. To prevent spurious multiple detections due to peg bouncing, sustained interactions, or events not related with physiologically meaningful motor actions, inhibition times of 100 ms (holes) and 200 ms (tray) are applied. Event timestamps are generated using an onboard 32 MHz oscillator (±10 ppm), corresponding to a negligible timing drift (≈±600 µs/min) relative to the temporal resolution required for the analysis.

The system therefore detects the instant the user interacts with a peg in the tray, correctly inserts it into a hole, and subsequently removes it, automatically recording the time taken for each of these actions, thus enabling the calculation of parameters beyond the total time required to complete the test and making the measurement assessor-independent.

A custom Android smartphone/tablet application is used to manage the database of tested individuals. The application connects to the device via Bluetooth and logs detailed movement data from peg insertion and removal. Custom software allows visualization and verification of record quality, including automatic error detection (e.g., missed insertions highlighted in yellow). It enables raw data acquisition and supports the computation of various temporal measures (e.g., the execution times for the peg insertion and removal phases are considered separately, as well as the time intervals between the insertion of one peg and the next). See Section 2.4 for further description of the new parameters.

### 2.3. Experimental Procedure

The participants were required to sit at a table, and the assessor provided standardized instructions before the first test.

The experimenter explained to the participants that they have to pick up each peg, one at a time, and insert it into a hole (Insertion phase). Then, they have to remove the pegs, one at a time, and place them back in the container (Removal phase). Both actions should be performed as quickly as possible. Only the tested hand may touch pegs; the hand not being tested can stabilize the board if needed, using a designated handle.

After a brief familiarization phase, the participants performed two trials with the dominant hand and two with the non-dominant hand, resulting in four trials. The dominant hand was always tested first. Manual stopwatch and automated measurements were recorded simultaneously.

To assess the reproducibility of the automated measures, data acquired automatically from the sensorized pegboard were processed via the app and compared to manual stopwatch recordings taken by a single expert assessor. The sensorized 9-HPT was activated, then it started recording automatically from the moment the fingers touched the first peg (they were in proximity of the peg, with an error less than 5 mm) until the last peg was placed back. The assessor had to start the stopwatch as the participant touched the first peg and stopped it when the last peg was placed back in the container. The execution times for each trial were recorded in seconds (s), and the average execution times across the two trials were calculated for each hand.

For the test–retest assessment, participants repeated the acquisition session twice, 1–2 weeks apart.

### 2.4. Sensorized 9-HPT—Parameter Description

The system can detect the following events: peg insertion, peg extraction, and tray interaction. Each event is time-stamped with a relative time starting from the start of the experiment.

*Total Time (TT)*—The main parameter recorded by the sensorized 9-HPT is the total time (TT) required to complete the task. The system automatically starts calculating the time when it detects that the first peg inside the tray is touched and stops when the last peg is placed in the tray. TT is reported in seconds.

*Insertion Time (IT)*—The insertion time refers to the time required for the participant to insert all 9 pegs into the 9 holes before the return phase begins. It starts when the first peg is touched and ends when the last hole is filled. It is a component of the TT and is reported in seconds.

*Removal Time (RT)*—The removal time refers to the time taken by the participant to extract all the 9 pegs from the holes and place them back in the dish. It starts when the first peg is extracted and ends when the last peg is placed in the tray. It is also part of the TT and is reported in seconds.

*Pause*—This parameter refers to the time interval between the IT and the RT, corresponding to the transition phase after all pegs have been inserted and before the removal phase begins. Specifically, it represents the time elapsed between the last peg insertion and the first peg removal.

It must be noted that:

(1) IT+RT+Pause=TT

*Mean Inter-Peg Interval (MIP)*—The device calculates the interval between two subsequent peg insertions and extractions. The inter-peg (IP) time represents the average time of the interaction between one peg and the following one. The MIP is reported in seconds.

*Inter-Peg Insertion (IP-I)*—This parameter refers to the inter-peg interval of the insertion phase, namely, the average time between each peg insertion and the following. IP-I is reported in seconds.

*Inter-Peg Removal (IP-R)*—This parameter refers to the inter-peg interval of the removal phase, namely, the average time between each peg extraction and the following. IP-R is reported in seconds.

To account for non-ideal execution, specific logic rules were implemented to manage short-term inconsistencies. If the peg bounces back during the insertion, rapid sequences such as peg insertion–removal–reinsertion within 100 ms are treated as a single insertion event to avoid artifacts. Similarly, consecutive tray interaction events (e.g., peg dropped and returned to the tray) are consolidated, and only the first event in an interrupted sequence is considered for the interval times computation.

### 2.5. Data Analysis

To assess the association between the total time measured by the assessor and by the sensorized 9-HPT system, correlation analyses were performed separately for each of the four trials. This approach was adopted to avoid artificial inflation of correlation estimates due to the non-independence of repeated measurements within participants. For each trial, either Pearson’s correlation coefficient (for normally distributed data) or Spearman’s rank correlation coefficient (otherwise) was computed to evaluate the strength of the association between the two measurements. The normality of the distributions of variables was assessed using the Shapiro–Wilk test.

In addition to correlation analysis, a linear mixed-effects model was fitted to account for repeated measurements within subjects. Subjects were included as a random effect, whereas method (automatic vs. rater), hand (dominant vs. non-dominant), and trial (1–4) were included as fixed effects. The model was specified as:

(2) time∼method×trial×hand+(1∣subj)

A Bland–Altman analysis was performed to assess the agreement between the two measurement methods. Given the absence of significant interaction effects among method, hand, and trial, all observations were pooled into a single Bland–Altman plot. Limits of agreement were calculated as bias ±1.96 × SD of the paired differences. Although a few outliers resulted in a distribution of differences that was not perfectly normal, the standard Bland–Altman approach was retained because these observations did not materially affect the estimated bias or the overall limits of agreement.

The test–retest reliability of the automated parameters recorded by the sensorized system was evaluated using ICCs. ICCs were calculated based on a two-way mixed-effects model with absolute agreement for the average of measurements [29]. Reliability analyses were performed separately for the dominant and non-dominant sides.

## 3. Results

Thirty-six participants (age M ± SD: 38.19 ± 11.28, 24 female) completed the assessment.

**Figure 2:**
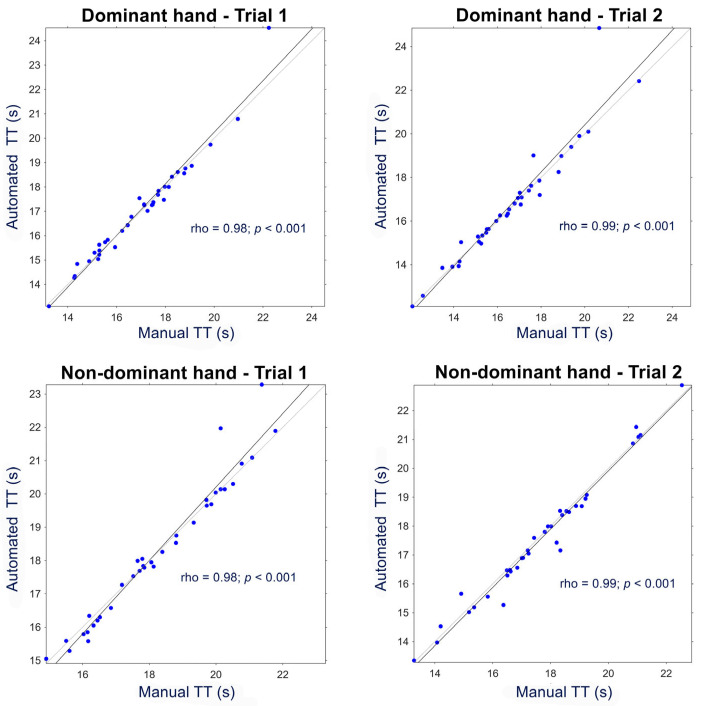
Scatter plots of rater manually measured (stopwatch) versus automatically recorded (sensorized 9-HPT) total times (TT) for the four trials (two performed with the dominant hand, namely D1 and D2, and two with the non-dominant hand, namely ND1 and ND2). Dotted line represents the bisector, and continuous line represents the fit.

Correlation analyses showed a strong association between the total time recorded by the assessor and the sensorized 9-HPT across all trials. Spearman’s rank correlation coefficient was used for trials performed with the dominant hand (D1–D2), whereas Pearson’s correlation coefficient was applied for trials performed with the non-dominant hand (ND1–ND2), based on the distribution of the data (see Figure 2). In particular, positive correlations between standard and automated measures for D1 (rho = 0.98), D2 (rho = 0.99), ND1 (rho = 0.98), and ND2 (rho = 0.99) have been found (*p* always < 0.001).

Linear mixed-effects model revealed a significant effect of hand, with longer execution times for the non-dominant hand (β = 2.74, SE = 1.01, t = 2.72, *p* = 0.007). No significant effect of measurement method was observed, indicating no systematic bias between the sensorized system and the rater. No significant main effect of trial or interaction effects were found among method, hand, and trial.

**Figure 3:**
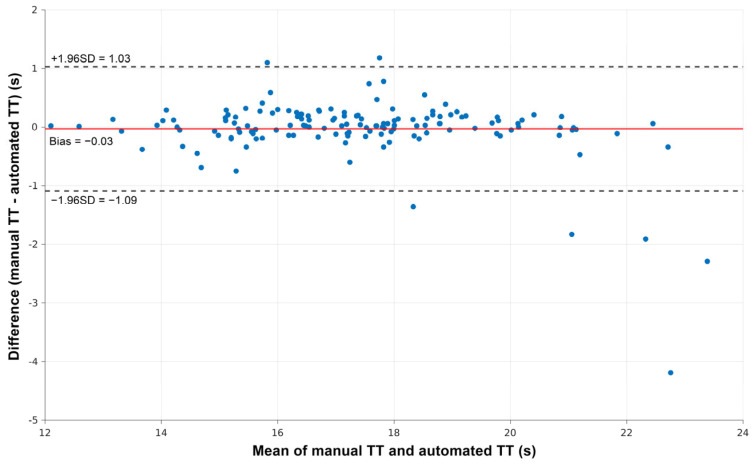
Bland–Altman plot comparing manual stopwatch-based measurements and automated measurements (total time—TT) recorded by the sensorized platform. The red line represents the estimated mean difference between the two measurements on the same participant. The two dashed lines indicate the limits of agreement (LOA).

The Bland–Altman analysis showed a mean bias of −0.03, with 95% limits of agreement (LOA) ranging from −1.09 to 1.03 (Figure 3), corresponding to approximately ±6% of the mean task duration [32].

The automated TT showed good test–retest reliability for both the dominant and non-dominant hands, with ICC values generally exceeding the predefined acceptability threshold of 0.50 (*p* always less than 0.05). Furthermore, when the task was executed with the dominant hand, the new parameter showed a moderate-to-good reliability ranging from 0.68 to 0.89. With the non-dominant hand, ICCs were generally more variable, highlighting a good-to-moderate reliability for all the parameters, except for the pause time, which showed a poor reliability. Table 1 shows the averaged data in the two sessions and ICC with confidence intervals and *p*-values.

## 4. Discussion

The study aimed to evaluate the psychometric characteristics of a sensorized version of the 9-HPT in a healthy population to assess the variability and reliability of the automated measurement thanks to the novel device.

Thirty-six healthy participants performed the 9-HPT on the sensorized board twice with each hand (dominant and non-dominant), and the concurrent validity was evaluated to assess the consistency between the expert assessor and the sensorized 9-HPT. Additionally, test–retest reliability was assessed in two sessions separated by 1–2 weeks.

Once its concurrent validity and the test–retest reliability had been verified, the next objective was to describe the new parameters that we were able to collect thanks to the sensorized platform and the associated app.

To assess the consistency between the expert assessor and the sensorized 9-HPT, a correlation was run between the total time calculated manually by the assessor, measured with a stopwatch, and that measured automatically by the sensorized system.

Overall, the concurrent validity between the measurements was consistently very strong, showing an excellent correlation between manual and automated measurements for both the dominant and non-dominant hands in all trials.

The Bland–Altman analyses showed a negligible mean bias (−0.03 s) between the two measurement methods, with LOA (−1.09 to 1.03 s) corresponding to approximately ±6% of the mean task duration. Furthermore, linear mixed-effects model supports the absence of systematic differences between methods, while showing a difference in the execution time between the dominant and non-dominant hands. Variability in upper limb function and deficits in fine motor control might amplify discrepancies between device-based and operator-based timing, and this aspect should be specifically investigated in future studies involving clinical populations. Nevertheless, it is worth noting that, in neurological conditions such as multiple sclerosis, changes in 9-HPT performance on the order of 20–30% are often considered clinically meaningful [33]. Therefore, the magnitude of disagreement observed in the present study is smaller than commonly reported thresholds for clinically important change, although its actual impact on clinical interpretation remains to be determined.

For the TT, the sensorized 9-HPT showed reliable outcomes over 2 weeks, with ICC ranging from 0.74 for the non-dominant hand to 0.81 for the dominant hand.

We further tested the test–retest reliability for the other kinematic parameters collected with the sensorized 9-HPT. The dominant hand showed ICC ranging from moderate to good between the two sessions, with particularly good reliability for the pause (ICC = 0.89). Interestingly, the new parameters recorded during the performances of the non-dominant hand showed a more variable reliability, highlighting a good-to-moderate reliability for all the parameters, except for the pause time, which showed a poor reliability; higher ICCs were found for the removal time (ICC = 0.77) and the inter-peg removal (ICC = 0.76), suggesting good consistency in repeated tests for the removal phase.

Although our results show reasonable reliability of the sensorized 9-HPT for both hands used to perform the test, in the literature, there is no complete agreement on the reliability of the TT with regard to the hand used or the different populations performing the test. In fact, strong reproducibility of the data over time, both in healthy populations and populations with various types of diseases, was reported [1,2], but also examples of better reliability of one hand rather than the other. For example, Mathiowez and colleagues [34] found an ICC of 0.69 for the dominant hand versus 0.43 for the non-dominant hand. In addition, Watanabe and colleagues tested the non-dominant hand in healthy participants, finding an ICC of 0.49, compared to both hands in patients with stroke, with an ICC always greater than 0.91 [35].

It has been suggested that reliability may be influenced by a learning effect resulting from test repetition [11,36]. This could be even more pronounced in the non-dominant hand, particularly in healthy participants, who may need a few trials to find the right motor strategy. However, only the dominant hand is often evaluated when testing the reliability of the 9-HPT [22,25,37].

Overall, these results suggest a good reliability of the sensorized 9-HPT, at least for the classic measurement of task completion time and for dominant hand performance. Further research is needed on the non-dominant hand and on learning effects.

The 9-HPT is a well-established test, whose compact design and minimal equipment requirements have contributed to its widespread use: it does not require complex equipment, it is lightweight and portable, and all these features make it well suited for use in clinics. However, several studies have proposed different versions of 9-HPT, ranging from full-digital iPad-based tools [20], to virtual reality [21], to actual sensorized devices [24,25,26] designed to replicate the original 9-HPT. In any case, each of these has demonstrated the tool’s ability to reliably reproduce the experimenter’s measurement and to have good test–retest reliability. However, the total time remains the standard outcome measure in the 9-HPT, but there is growing interest in identifying additional parameters to better capture manual dexterity impairments in people with multiple sclerosis. A very recent study proposed novel digital features, such as hand movement speed, showing improved reliability and prognostic value over time alone [22]. This approach was based on a fully digital version of the 9-HPT, which does not require actual reaching, pinching, grasping and manipulation of physical pegs, thus limiting its ability to assess fine motor control in an ecologically valid way. This study highlights the need and opportunity to develop enhanced sensor-based 9-HPT platforms that preserve the structure of the original test while enabling the extraction of new, clinically meaningful metrics. However, it has been suggested on several occasions that the 9-HPT completion score alone may be limiting. For example, Angelucci and colleagues pointed out that total time is not descriptive of how a participant performs the task, because slow performance could be indicative of greater accuracy or greater difficulty [14]. For this reason, some researchers have started to explore the possibility of employing other measurements for both temporal and spatial kinematic parameters and for the possibility of recognizing a strategy. Johannson and colleagues used a complex system of optical cameras to record the kinematics of the upper limbs during the execution of a custom-made 9-HPT, in addition to manipulating the order of peg insertion in stroke patients, to better describe temporal characteristics through spatial ones [23].

Fan and colleagues dissected the video-recorded movement into different phases, including the grasping of the peg, the speed peaks of the movement, etc., suggesting that each of these aspects may be linked to a different motor function, ranging from flexibility to smoothness to efficiency [24]. Various studies have proposed video analysis to detect new parameters [23,24,37].

In this study, we have proposed a version resembling the classical 9-HPT that does not require additional preparation of the participant by the clinician, but is able to detect kinematic parameters while remaining a compact device. Some of these parameters have proven to be more reliable than others; however, it should be noted that our sample consisted exclusively of healthy participants, whereas the 9-HPT is most effective in a clinical setting.

Furthermore, the present study focused on temporal parameters; however, the sensorized platform enables the recording of peg insertion sequences, facilitating future exploration of individual motor planning strategies and a novel pathway to investigate the cognitive dimensions of manual dexterity, which remain largely unexplored in clinical populations. The proposed temporal metrics may provide a more detailed characterization of upper limb dexterity by separately quantifying distinct phases of the task. Such parameters could help identify subtle alterations in motor planning, movement execution, grasp–release coordination, or movement variability that may not be detectable through TT alone. In clinical settings, these metrics could therefore support a more sensitive assessment of motor impairment progression and rehabilitation outcomes, particularly in neurological conditions affecting fine motor control. However, their clinical relevance and sensitivity will require further investigation in patient populations.

The primary aim of this study was to introduce a new sensorized 9-HPT platform, describe its technical capabilities, and propose novel temporal parameters. To this end, the system was evaluated in a cohort of healthy participants without upper limb impairments, who are expected to show relatively low variability in task execution. Within this context, the results appear promising in terms of technical performance, agreement with standard clinical timing methods, and test–retest reliability. However, several limitations should be acknowledged. Although the study demonstrates the technical validity of the sensorized 9-HPT, the validation is limited to timing accuracy and reliability in healthy individuals. The novel temporal parameters provided by the device were not validated against an independent external reference; therefore, their construct and clinical validity remain to be established. In particular, their clinical relevance cannot be inferred from the present data, as this would require evaluation in clinical populations and assessment of their association with meaningful functional outcomes. In addition, the robustness of the logic used to consider the start of the recording and to prevent spurious multiple detections (e.g., signal bouncing or repeated insertions) in populations with impaired dexterity, has not yet been verified.

## 5. Conclusions

To the best of our knowledge, the sensorized 9-HPT presented here represents a promising comprehensive tool capable of automating the execution of the test and of detecting new parameters while maintaining the appearance of commercial 9-HPT without adding bulky instrumentation, such as cameras.

The application of the sensorized 9-HPT to neurological populations to explore the clinical validity of the proposed parameters and assess whether they can serve as biomarkers of pathology is a topic for future research. Importantly, comprehensive normative reference data of healthy individuals, stratified by age and gender, are needed to provide a more refined framework for evaluating manual dexterity across the lifespan, including the newly introduced automated metrics. This will ensure robust estimation of the standard error of measurement and the minimal detectable change in the proposed parameters. Furthermore, validation of the sensorized 9-HPT in clinical populations with impaired manual dexterity will be essential to determine the clinical validity and utility of the device, as well as the potential added value of the newly introduced parameters.

Finally, further development of the app will be necessary to enhance its usability and clarity, ensuring that the interface is intuitive and easily interpretable for both clinicians and patients, thereby facilitating its integration into routine clinical practice.

## Figures and Tables

**Table 1:** Descriptive statistics of 9-HPT parameters and Intraclass correlation coefficient (ICC) results. Session 1 and Session 2 columns represent the median of the averaged measurements across trials 1 and 2 and interquartile range (IQR) reported in seconds, separately for the dominant and non-dominant hand (D and ND, respectively). ICC results are presented with 95% confidence intervals and *p*-values. The Level of Reliability column reports the interpretation of ICC values according to the criteria proposed by Koo and Li [29].

Hand	Measurement	Session 1	Session 2	ICC (95% CI)	Level of Reliability	*p* Value
Dominant	TT	16.72 [15.36–17.92]	16.15 [15.21–16.67]	0.81 (0.60–0.90)	Good	* 0.006
	IT	11.17 [9.97–11.80]	10.53 [9.84–11.11]	0.74 (0.48–0.87)	Moderate	* 0.034
	RT	4.66 [4.46–5.11]	4.66 [4.44–5.19]	0.76 (0.54–0.88)	Good	* 0.014
	Pause	0.94 [0.87–1.02]	0.85 [0.77–0.95]	0.89 (0.66–0.95)	Good	* 0.005
	MIP	0.95 [0.89–1.00]	0.92 [0.86–0.94]	0.68 (0.38–0.84)	Moderate	0.090
	IP-I	1.34 [1.19–1.41]	1.24 [1.18–1.32]	0.78 (0.56–0.89)	Good	* 0.010
	IP-R	0.56 [0.53–0.61]	0.56 [0.53–0.63]	0.73 (0.48–0.86)	Moderate	* 0.034
Non-Dominant	TT	17.84 [16.38–19.13]	17.64 [16.58–18.51]	0.74 (0.50–0.87)	Moderate	* 0.025
	IT	11.61 [11.03–12.84]	11.48 [11.08–12.06]	0.57 (0.17–0.78)	Moderate	0.319
	RT	5.03 [4.59–5.37]	5.00 [4.64–5.51]	0.77 (0.54–0.88)	Good	* 0.013
	Pause	0.92 [0.75–1.09]	0.91 [0.83–1.06]	0.42 (−0.15–0.71)	Poor	0.665
	MIP	1.01 [0.94–1.09]	1.00 [0.94–1.03]	0.68 (0.37–0.83)	Moderate	0.100
	IP-I	1.39 [1.31–1.53]	1.38 [1.31–1.44]	0.56 (0.14–0.77)	Moderate	0.356
	IP-R	0.60 [0.55–0.65]	0.60 [0.56–0.66]	0.76 (0.52–0.88)	Good	* 0.018

* statistically significant results.

## Data Availability

The raw data supporting the conclusions of this article will be made available by the authors on request.

## Abbreviations

The following abbreviations are used in this manuscript:

9-HPT	Nine-Hole Peg Test
